# Supplementary material for: Shortness of breath in children at the emergency department: Variability in management in Europe
Source: PLoS One. 2021 May 5;16(5):e0251046. doi: 10.1371/journal.pone.0251046 (PMC8099081; doi:10.1371/journal.pone.0251046)
Supplement: S1 Table — (PDF) [file pone.0251046.s001.pdf]

**S1 Table. Description of the different study sites**

| | Erasmus MC, Rotterdam, the Netherlands Hospital A | Maasstad Hospital, Rotterdam, the Netherlands Hospital B | St Mary's Hospital, London, United Kingdom Hospital C | Hospital Fernando da Fonseca, Lisbon, Portugal Hospital D | General Hospital, Vienna, Austria Hospital E |
|---|---|---|---|---|---|
| Hospital characteristics | University hospital<br>60 paediatric beds | Teaching hospital<br>59 paediatric beds | University hospital<br>46 paediatric beds | Community hospital<br>91 paediatric beds | University hospital<br>74 paediatric beds |
| Catchment area | Urban<br><br>Mixed high and low socio-economic status | Urban<br><br>Generally low socio-economic status | Urban<br><br>Mixed high and low socio-economic status | Mixed urban and rural<br>Generally low socio-economic status | Urban<br><br>Mixed high and low socio-economic status |
| Emergency department characteristics | Paediatric only<br><br>6500 children/year | Mixed adult-paediatric<br><br>9500 children/year | Paediatric only<br><br>27.000 children/year | Paediatric only<br><br>60.000 children/year | Paediatric only<br><br>22.000 children/year |
| Supervising physician | Paediatrician | Paediatrician | Paediatric emergency physician | Paediatrician | Paediatrician |
| Inclusion period | 01-01-2012 to 31-12-2014 | 01-05-2014 to 31-10-2015 | 01-07-2014 to 28-02-2015 | 01-03-2014 to 28-02-2015 | 01-01-2014 to 31-12-2014 |
| Number of patients included | 1.558 | 1.268 | 1.639 | 5.729 | 3.358 |
| Primary care availability | 24/7 | 24/7 | Daytime and evenings | Daytime and evenings | Daytime |
| Referral by emergency service | 8.9% | 4.5% | 5.6% | 4.0% | Not available |
| Self-referral | 27% | 17% | 82% | 96% | >90%* |
| Comorbidity in all children | 38% | <10%* | 11% | <10%* | 10% |
| Comorbidity in children with shortness of breath | 54% | < 10%* | 18% | < 10%* | 11% |

* overall data for this setting, not available for each individual patient
