# Supplementary material for: Shortness of breath in children at the emergency department: Variability in management in Europe
Source: PLoS One. 2021 May 5;16(5):e0251046. doi: 10.1371/journal.pone.0251046 (PMC8099081; doi:10.1371/journal.pone.0251046)
Supplement: S2 Table — (PDF) [file pone.0251046.s002.pdf]

S2 Table. Immediate life-saving interventions

1. Airway and breathing support, including intubation or emergent noninvasive positive pressure ventilation.
2. Electrical therapy, including defibrillation, emergent cardioversion, or external pacing.
3. Procedures, including chest needle decompression, pericardiocentesis, or open thoracotomy.
4. Hemodynamic support, including significant intravenous fluid resuscitation in the setting of hypotension, blood administration, or control of major bleeding.
5. Emergency medications, including naloxone, dextrose, atropine, adenosine, epinephrine, or vasopressors
