# Supplementary material for: Shortness of breath in children at the emergency department: Variability in management in Europe
Source: PLoS One. 2021 May 5;16(5):e0251046. doi: 10.1371/journal.pone.0251046 (PMC8099081; doi:10.1371/journal.pone.0251046)
Supplement: S3 Table — (PDF) [file pone.0251046.s003.pdf]

S3 Table. Differences in diagnostic tests between EDs

S3a. Differences in diagnostic tests between ED's: all children#

	Blood tests Adjusted OR	95% CI	X-rays Adjusted OR	95% CI
NL tertiary	3.9*	3.1-4.8	5.0*	3.9-6.4
NL teaching	1.2**	0.9-1.6	Reference	-
UK	Reference	-	2.3*	1.8-3.0
PT	1.4*	1.1-1.7	8.8*	7.0-11.1
AT	5.3*	4.3-6.6	4.1*	3.2-5.3

S3b. Children younger than 1 year#

	Blood tests Adjusted OR	95% CI	X-rays Adjusted OR	95% CI
NL tertiary	3.4*	2.2-5.2	11.6*	6.1-22.0
NL teaching	1.2**	0.7-1.9	Reference	-
UK	Reference	-	5.6*	2.8-11.1
PT	1.8*	1.2-2.7	17.7*	9.6-32.7
AT	5.8*	3.8-8.8	11.2*	5.8-21.7

S3c. Children older than 1 year#

	Blood tests Adjusted OR	95% CI	X-rays Adjusted OR	95% CI
NL tertiary	4.1*	3.1-5.3	4.0*	3.1-5.3
NL teaching	1.3**	0.9-1.7	Reference	-
UK	Reference	-	1.9*	1.4-2.6
PT	1.2**	0.9-1.6	7.5*	5.8-9.7
AT	5.1*	3.9-6.6	3.3*	2.5-4.3

S3d. Children with a severe presentation#

	Blood tests Adjusted OR	95% CI	X-rays Adjusted OR	95% CI
NL tertiary	3.6*	2.8-4.6	4.7*	3.6-6.2
NL teaching	Reference	-	Reference	-
UK	1.0**	0.8-1.3	2.3*	1.7-3.1
PT	1.3**	1.0-1.6	8.0*	6.2-10.4
AT	5.6*	4.2-7.3	4.3*	3.2-5.8

S3e. Children with a non-severe presentation#

	Blood tests Adjusted OR	95% CI	X-rays Adjusted OR	95% CI
NL tertiary	14.3*	5.9-34.7	9.1*	2.5-33.1
NL teaching	4.7*	1.8-12.4	Reference	-
UK	Reference	-	2.3**	0.6-8.5
PT	4.3*	1.8-10.1	21.0*	6.1-72.6
AT	11.7*	5.1-26.6	7.3*	2.1-25.1

#Associations are determined by multivariable logistic regression models. Model adjusted for sex, age, season, triage urgency, fever, tachycardia, tachypnoea, low oxygen saturation and increased work of breathing.
* P-value <0.001. ** not significant
NL teaching = Maasstad Hospital, Rotterdam, the Netherlands; NL tertiary = Erasmus MC, Rotterdam, the Netherlands; UK = St Mary's Hospital, London, United Kingdom; PT = Hospital Fernando da Fonseca, Lisbon, Portugal; AT = General Hospital, Vienna, Austria.
