# Supplementary material for: Shortness of breath in children at the emergency department: Variability in management in Europe
Source: PLoS One. 2021 May 5;16(5):e0251046. doi: 10.1371/journal.pone.0251046 (PMC8099081; doi:10.1371/journal.pone.0251046)
Supplement: S4 Table — (PDF) [file pone.0251046.s004.pdf]

S4 Table. Differences in medication between EDs

S4a. Differences in medication between ED's: all children#

	Inhalation medication Adjusted OR	95% CI	Intravenous medication Adjusted OR	95% CI
NL tertiary	1.1**	1.0-1.3	2.0*	1.5-2.6
NL teaching	1.6*	1.3-1.8	4.1*	3.1-5.5
UK	1.7*	1.5-2.0	Reference	-
PT	2.2*	1.9-2-5	1.3**	1.0-1.7
AT	Reference	0.5-0.8	1.1**	0.8-1.5

S4b. Differences in medication between ED's in children younger than 1 year#

	Inhalation medication Adjusted OR	95% CI	Intravenous medication Adjusted OR	95% CI
NL tertiary	1.2**	0.9.-1.6	2.5*	1.4-4.5
NL teaching	1.8*	1.3-2.5	3.7*	2.1-6.7
UK	Reference	-	Reference	-
PT	3.0*	2.3-3.9	1.3**	0.8-2.3
AT	1.8*	1.3-2.4	1.6**	0.8-3.1

S4c. Differences in medication between ED's in children older than 1 year#

	Inhalation medication Adjusted OR	95% CI	Intravenous medication Adjusted OR	95% CI
NL tertiary	1.3*	1.1-1.6	1.7*	1.2-2.4
NL teaching	1.8*	1.5-2.2	4.4*	3.2-6.0
UK	2.4*	2.1-2.9	1.0**	0.7-1.4
PT	2.4*	2.1-2.7	1.2**	0.9-1.7
AT	Reference	-	Reference	-

S4d. Children with a severe presentation#

	Inhalation medication Adjusted OR	95% CI	Intravenous medication Adjusted OR	95% CI
NL tertiary	Reference	-	1.9*	1.4-2.3
NL teaching	1.5*	1.2-1.8	3.0*	2.3-3.8
UK	1.7*	1.4-2.1	1.0**	0.7-1.3
PT	2.0*	1.7-2.4	Reference	-
AT	1.0**	0.8-1.2	1.6*	1.2-2.1

S4e. Children with a non-severe presentation#

	Inhalation medication Adjusted OR	95% CI	Intravenous medication Adjusted OR	95% CI
NL tertiary	Reference	-	9.9*	1.2-79.4
NL teaching	2.9*	1.5-5.5	65.8*	8.9-487.3
UK	2.0*	1.1-3.6	Reference	-
PT	4.6*	2.7-7.9	15.1*	2.1-106.9
AT	1.8*	1.1-3.1	4.3**	0.6-31.8

#Associations are determined by multivariable logistic regression models. Model adjusted for sex, age, season, triage urgency, fever, tachycardia, tachypnoea, low oxygen saturation and increased work of breathing.

* P-value <0.001. ** not significant

NL teaching = Maasstad Hospital, Rotterdam, the Netherlands; NL tertiary = Erasmus MC, Rotterdam, the Netherlands; UK = St Mary's Hospital, London, United Kingdom; PT = Hospital Fernando da Fonseca, Lisbon, Portugal; AT = General Hospital, Vienna, Austria.
