# Supplementary material for: Shortness of breath in children at the emergency department: Variability in management in Europe
Source: PLoS One. 2021 May 5;16(5):e0251046. doi: 10.1371/journal.pone.0251046 (PMC8099081; doi:10.1371/journal.pone.0251046)
Supplement: S5 Table — (PDF) [file pone.0251046.s005.pdf]

## S5 Table. Differences in hospital admission between EDs

### S5a. Differences in hospital admission between ED's: all children#

| | Hospital admission Adjusted OR | 95% CI | PICU admission Adjusted OR | 95% CI |
|---|---|---|---|---|
| NL tertiary | 9.8* | 8.3-11.5 | 75.8* | 18.5-310.7 |
| NL teaching | 7.6* | 6.4-9.1 | 1.7** | 0.3-8.9 |
| UK | 4.0* | 3.4-4.8 | 2.6** | 0.5-12.6 |
| PT | Reference | - | 8.5* | 2.0-35.9 |
| AT | 2.2* | 1.8-2.6 | Reference | - |

### S5b. Differences in hospital admission between ED's in children younger than 1 year#

| | Hospital admission Adjusted OR * | 95% CI | PICU admission Adjusted OR* | 95% CI |
|---|---|---|---|---|
| NL tertiary | 6.6* | 5.1-8.7 | 78.0* | 10.5-579.8 |
| NL teaching | 4.6* | 3.5-6.0 | Reference | - |
| UK | 1.4* | 1.0-1.9 | 1.2** | 0.1-20.2 |
| PT | Reference | - | 15.9* | 2.1-121.0 |
| AT | 1.4* | 1.0-2.0 | 1.8** | 0.1-30.5 |

### S5c. Differences in hospital admission between ED's in children older than 1 year#

| | Hospital admission Adjusted OR * | 95% CI | PICU admission Adjusted OR* | 95% CI |
|---|---|---|---|---|
| NL tertiary | 12.6* | 10.2-15.5 | 117.7* | 16.1-860.9 |
| NL teaching | 11.1* | 8.7-14.0 | 3.4** | 0.4-31.1 |
| UK | 6.8* | 5.5-8.4 | 4.9** | 0.6-42.5 |
| PT | Reference | - | 7.1** | 0.9-54.8 |
| AT | 2.9* | 2.3-3.6 | Reference | - |

### S5d. Children with a severe presentation#

| | Hospital admission Adjusted OR * | 95% CI | PICU admission Adjusted OR* | 95% CI |
|---|---|---|---|---|
| NL tertiary | 10.0* | 8.3-12.0 | 52.2* | 12.7-213.9 |
| NL teaching | 6.9* | 5.6-8.4 | 1.4** | 0.3-7.3 |
| UK | 4.1* | 3.4-4.9 | 2.0** | 0.4-9.9 |
| PT | Reference | - | 6.2* | 1.5-26.2 |
| AT | 2.4* | 1.9-3.1 | Reference | - |

### S5e. Children with a non-severe presentation#

| | Hospital admission Adjusted OR * | 95% CI | PICU admission Adjusted OR* | 95% CI |
|---|---|---|---|---|
| NL tertiary | 10.7* | 5.4-21.2 | n.a. | - |
| NL teaching | 12.6* | 6.1-26.3 | n.a. | - |
| UK | 1.9** | 0.8-4.4 | n.a. | - |
| PT | Reference | - | n.a. | - |
| AT | 1.6** | 0.8-3.1 | n.a. | - |

#Associations are determined by multivariable logistic regression models. Model adjusted for sex, age, season, triage urgency, fever, tachycardia, tachypnoea, low oxygen saturation and increased work of breathing.

* P-value <0.001. ** not significant

NL teaching = Maasstad Hospital, Rotterdam, the Netherlands; NL tertiary = Erasmus MC, Rotterdam, the Netherlands; UK = St Mary's Hospital, London, United Kingdom; PT = Hospital Fernando da Fonseca, Lisbon, Portugal; AT = General Hospital, Vienna, Austria.
