# Supplementary material for: Shortness of breath in children at the emergency department: Variability in management in Europe
Source: PLoS One. 2021 May 5;16(5):e0251046. doi: 10.1371/journal.pone.0251046 (PMC8099081; doi:10.1371/journal.pone.0251046)
Supplement: S6 Table — (PDF) [file pone.0251046.s006.pdf]

S6 Table. Differences in resource use in children with and without comorbidity.

	No comorbidity<br>N (%)<br>5045	Non-complex comorbidity<br>N (%)<br>817	Complex comorbidity<br>N (%)<br>693	P-value
Blood tests	796 (15.8)	144 (17.6)	309 (44.6)	<0.001
X-rays	680 (13.5)	156 (19.1)	239 (34.5)	<0.001
Inhalation medication	1463 (29.0)	428 (52.4)	213 (30.7)	<0.001
Intravenous medication	219 (4.3)	58 (7.1)	102 (14.7)	<0.001
General admission	790 (15.7)	241 (29.5)	312 (45.0)	<0.001
ICU admission	49 (1.0)	26 (3.2)	71 (10.2)	<0.001
