# Supplementary material for: Shortness of breath in children at the emergency department: Variability in management in Europe
Source: PLoS One. 2021 May 5;16(5):e0251046. doi: 10.1371/journal.pone.0251046 (PMC8099081; doi:10.1371/journal.pone.0251046)
Supplement: S7 Table — (PDF) [file pone.0251046.s007.pdf]

S7 Table. Heatmap with odds ratios of resource use, excluding patients with comorbidity.

S7a. Heatmap for different ages with odds ratios of resource use, corrected for patient characteristics[#]

	NL tertiary	NL teaching	UK	PT	AT
Blood tests all children	3.5*	1.4*	+	1.6*	4.9*
< 1 year	3.7*	1.4**	+	2.2*	5.5*
> 1 year	3.7*	1.4**	+	1.3**	4.6*
X-rays all children	4.4*	+	2.2*	8.6*	3.6*
< 1 year	11.4*	+	4.4*	18.2*	8.3*
> 1 year	3.2*	+	1.9*	7.3*	2.9*
Inhalation medication all children	1.0**	1.5*	1.5*	2.1*	+
< 1 year	1.2**	1.8*	+	2.9*	1.9*
> 1 year	1.3*	1.7*	2.2*	2.3*	+
Intravenous medication all children	2.2*	5.7*	+	1.6*	1.2**
< 1 year	3.2*	5.0*	+	1.7**	1.8**
> 1 year	1.5**	6.1*	+	1.5*	1.1**
General admission all children	8.9*	8.0*	3.7*	+	1.8*
< 1 year	5.8*	4.6*	1.3**	+	1.1**
> 1 year	12.6*	12.4*	6.6*	+	2.7*
ICU admission all children	67.1*	2.2**	1.3**	10.6*	+
< 1 year	60.8*	+	0.0**	16.9*	0.0**
> 1 year	55.7*	1.8**	1.6**	3.8**	+

[#]Associations are determined by multivariable logistic regression models. Model adjusted for sex, age, season, triage urgency, fever, tachycardia, tachypnoea, low oxygen saturation and increased work of breathing.
[+] reference. * P-value <0.01. ** not significant
NL teaching = Maasstad Hospital, Rotterdam, the Netherlands; NL tertiary = Erasmus MC, Rotterdam, the Netherlands; UK = St Mary's Hospital, London, United Kingdom; PT = Hospital Fernando da Fonseca, Lisbon, Portugal; AT = General Hospital, Vienna, Austria.

S7b. Heatmap for patients with different severity with odds ratios of resource use, corrected for patient characteristics#

	NL tertiary	NL teaching	UK	PT	AT
Blood tests all children	3.5*	1.4*	+	1.6*	4.9*
severe	3.1*	1.1**	+	1.4*	5.2*
non-severe	13.8*	4.5*	+	4.1*	9.7*
X-rays all children	4.4*	+	2.2*	8.6*	3.6*
severe	4.3*	+	2.2*	7.8*	3.5*
non-severe	6.7*	+	1.8**	20.7*	6.6*
Inhalation medication all children	1.0**	1.5*	1.5*	2.1*	+
severe	+	1.4*	1.5*	1.9*	1.0**
non-severe	+	3.0*	1.5**	5.1*	1.9**
Intravenous medication all children	2.2*	5.7*	+	1.6*	1.2**
severe	2.2*	4.4*	+	1.3**	2.1*
non-severe	6.1**	56.7*	+	12.4*	3.1**
General admission all children	8.9*	8.0*	3.7*	+	1.8*
severe	9.0*	7.3*	4.0*	+	2.2*
non-severe	12.2*	14.6*	1.4**	+	1.3**
ICU admission all children	67.1*	2.2**	1.3**	10.6*	+
severe	53.2*	2.0**	1.1**	9.3*	+
non-severe	n.a.	n.a.	n.a.	n.a.	n.a.

#Associations are determined by multivariable logistic regression models. Model adjusted for sex, age, season, triage urgency, fever, tachycardia, tachypnoea, low oxygen saturation and increased work of breathing.
+ reference. * P-value <0.01. ** not significant
NL teaching = Maasstad Hospital, Rotterdam, the Netherlands; NL tertiary = Erasmus MC, Rotterdam, the Netherlands; UK = St Mary's Hospital, London, United Kingdom; PT = Hospital Fernando da Fonseca, Lisbon, Portugal; AT = General Hospital, Vienna, Austria.
