# Supplementary material for: Shortness of breath in children at the emergency department: Variability in management in Europe
Source: PLoS One. 2021 May 5;16(5):e0251046. doi: 10.1371/journal.pone.0251046 (PMC8099081; doi:10.1371/journal.pone.0251046)
Supplement: S8 Table — (PDF) [file pone.0251046.s008.pdf]

S8 Table. Heatmap with odds ratios of resource use for children with a past medical history of asthma.

	NL tertiary	UK	AT
Blood tests all children	4.0	*	14.4
X-rays all children	2.5	*	1.6**
Inhalation medication all children	1.9**	2.4	*
Intravenous medication all children	2.1**	*	4.2
General admission all children	2.1**	2.2**	*
ICU admission all children	**	**	**

[#]Associations are determined by multivariable logistic regression models. Model adjusted for sex, age, referral, season, triage urgency, fever, tachycardia, tachypnoea, low oxygen saturation and increased work of breathing.
[+] reference. * P-value <0.01. ** not significant
NL tertiary = Erasmus MC, Rotterdam, the Netherlands; UK = St Mary's Hospital, London, United Kingdom; AT = General Hospital, Vienna, Austria.
