# Supplementary material for: Shortness of breath in children at the emergency department: Variability in management in Europe
Source: PLoS One. 2021 May 5;16(5):e0251046. doi: 10.1371/journal.pone.0251046 (PMC8099081; doi:10.1371/journal.pone.0251046)
Supplement: S9 Table — (PDF) [file pone.0251046.s009.pdf]

**S9 Table. Heatmap with odds ratios of resource use, excluding patients from the ED in Austria.**

**S9a. Heatmap for different ages with odds ratios of resource use, corrected for patient characteristics[#]**

| | NL tertiary | NL teaching | UK | PT |
|---|---|---|---|---|
| Blood tests all children | 3.9* | 1.1** | + | 1.6* |
| < 1 year | 3.6* | 1.1** | + | 2.0* |
| > 1 year | 4.1* | 1.1** | + | 1.4* |
| X-rays all children | 5.2* | + | 2.4* | 9.7* |
| < 1 year | 11.8* | + | 5.8* | 18.6* |
| > 1 year | 4.3* | + | 2.0* | 8.4* |
| Inhalation medication all children | + | 1.5* | 1.6* | 2.0* |
| < 1 year | 1.1** | 1.7* | + | 2.8* |
| > 1 year | + | 1.4* | 1.9* | 1.8* |
| Intravenous medication all children | 2.1* | 4.3* | + | 1.4* |
| < 1 year | 2.6* | 3.8* | + | 1.4** |
| > 1 year | 1.8* | 4.6* | + | 1.4** |
| General admission all children | 9.2* | 7.2* | 3.9* | + |
| < 1 year | 6.4* | 4.5* | 1.4* | + |
| > 1 year | 11.7* | 10.4* | 6.5* | + |
| ICU admission all children | 44.7* | + | 1.5** | 5.0* |
| < 1 year | 78.0* | + | 1.2** | 15.8* |
| > 1 year | 34.5* | + | 1.5** | 2.1** |

[#]Associations are determined by multivariable logistic regression models. Model adjusted for sex, age, season, triage urgency, fever, tachycardia, tachypnoea, low oxygen saturation and increased work of breathing.

[+] reference. * P-value <0.01. ** not significant

NL teaching = Maasstad Hospital, Rotterdam, the Netherlands; NL tertiary = Erasmus MC, Rotterdam, the Netherlands; UK = St Mary's Hospital, London, United Kingdom; PT = Hospital Fernando da Fonseca, Lisbon, Portugal.

**S9b. Heatmap for patients with different severity with odds ratios of resource use, corrected for patient characteristics#**

| | NL tertiary | NL teaching | UK | PT |
|---|---|---|---|---|
| Blood tests all children | 3.9* | 1.1** | + | 1.6* |
| severe | 3.7* | + | 1.1** | 1.5* |
| non-severe | 13.5* | 4.3* | + | 4.4* |
| X-rays all children | 5.2* | + | 2.4* | 9.7* |
| severe | 4.8* | + | 2.4* | 8.7* |
| non-severe | 9.0* | + | 2.4** | 21.6* |
| Inhalation medication all children | + | 1.5* | 1.6* | 2.0* |
| severe | + | 1.5* | 1.8* | 2.1* |
| non-severe | + | 3.2* | 2.1* | 4.4* |
| Intravenous medication all children | 2.1* | 4.3* | + | 1.4* |
| severe | 1.9* | 3.2* | + | 1.1** |
| non-severe | 9.9* | 69.8* | + | 13.8* |
| General admission all children | 9.2* | 7.2* | 3.9* | + |
| severe | 9.2* | 6.4* | 4.0* | + |
| non-severe | 11.6* | 15.3* | 2.1** | + |
| ICU admission all children | 44.7* | + | 1.5** | 5.0* |
| severe | 37.5* | + | 1.5** | 4.5* |
| non-severe | n.a. | n.a. | n.a. | n.a. |

#Associations are determined by multivariable logistic regression models. Model adjusted for sex, age, season, triage urgency, fever, tachycardia, tachypnoea, low oxygen saturation and increased work of breathing.

+ reference.  * P-value <0.01. ** not significant

NL teaching = Maasstad Hospital, Rotterdam, the Netherlands; NL tertiary = Erasmus MC, Rotterdam, the Netherlands; UK = St Mary's Hospital, London, United Kingdom; PT = Hospital Fernando da Fonseca, Lisbon, Portugal.
