# Supplementary material for: Shortness of breath in children at the emergency department: Variability in management in Europe
Source: PLoS One. 2021 May 5;16(5):e0251046. doi: 10.1371/journal.pone.0251046 (PMC8099081; doi:10.1371/journal.pone.0251046)
Supplement: S10 Table — (PDF) [file pone.0251046.s010.pdf]

S10 Table. Heatmap with odds ratios of resource use, complete case analysis for respiratory rate.

S10a. Heatmap for different ages with odds ratios of resource use, corrected for patient characteristics#

	NL tertiary	NL teaching	UK	PT	AT
Blood tests all children	4.6	1.3**	*	1.8	10.2
< 1 year	4.1	1.2**	*	2.2	11.9
> 1 year	5	1.4**	*	1.6	9.7
X-rays all children	5.4	*	2.1	9.5	5.8
< 1 year	11.1	*	4.5	18.2	17.6
> 1 year	4.4	*	1.8	8.1	4.4
Inhalation medication all children	1.5	2.1	2.1	2.7	*
< 1 year	1.2**	1.8	*	2.7	1.2**
> 1 year	1.7	2.1	2.7	2.6	*
Intravenous medication all children	4.7	7.7	2.0**	3.2	*
< 1 year	2.7	3.0	*	1.5**	1.3**
> 1 year	5.2	10.6	2.4**	4.0	*
General admission all children	9.6	6.5	3.6	*	1.8
< 1 year	6.5	4.1	1.3**	*	1.5**
> 1 year	12.1	9.2	5.9	*	2.3
ICU admission all children	39.8	*	1.2**	4.3	2.2**
< 1 year	**	**	**	**	**
> 1 year	33.7	*	1.5**	2.2**	2.2**

#Associations are determined by multivariable logistic regression models. Model adjusted for sex, age, season, triage urgency, fever, tachycardia, tachypnoea, low oxygen saturation and increased work of breathing.

+reference. * P-value <0.01. ** not significant

NL teaching = Maasstad Hospital, Rotterdam, the Netherlands; NL tertiary = Erasmus MC, Rotterdam, the Netherlands; UK = St Mary's Hospital, London, United Kingdom; PT = Hospital Fernando da Fonseca, Lisbon, Portugal; AT = General Hospital, Vienna, Austria.

S10b. Heatmap for patients with different severity with odds ratios of resource use, corrected for patient characteristics#

	NL tertiary	NL teaching	UK	PT	AT
Blood tests all children	4.6	1.3**	*	1.8	10.2
severe	4.2	1.1**	*	1.6	8.4
non-severe	16.1	4	*	4.3	20.3
X-rays all children	5.4	*	2.1	9.5	5.8
severe	4.7	*	1.9	8.2	5.4
non-severe	4.6	0**	*	9.1	3.7
Inhalation medication all children	1.5	2.1	2.1	2.7	*
severe	1.1**	1.6**	1.8	2.1	*
non-severe	*	3.5	2.0	4.2	1.2**
Intravenous medication all children	4.7	7.7	2.0**	3.2	*
severe	3.1	4.2	1.4	1.8**	*
non-severe	15.8	81.4	*	18.3	2.5**
General admission all children	9.6	6.5	3.6	*	1.8
severe	9.7	6.0	3.7	*	1.5**
non-severe	13.0	11.7	2.2**	*	1.3**
ICU admission all children	39.8	*	1.2**	4.3	2.2**
severe	34.5	*	1.1**	4.0	3.1**
non-severe	n.a.	n.a.	n.a.	n.a.	n.a.

#Associations are determined by multivariable logistic regression models. Model adjusted for sex, age, season, triage urgency, fever, tachycardia, tachypnoea, low oxygen saturation and increased work of breathing.

+ reference. * P-value <0.01. ** not significant

NL teaching = Maasstad Hospital, Rotterdam, the Netherlands; NL tertiary = Erasmus MC, Rotterdam, the Netherlands; UK = St Mary's Hospital, London, United Kingdom; PT = Hospital Fernando da Fonseca, Lisbon, Portugal; AT = General Hospital, Vienna, Austria.
