# Supplementary material for: Shortness of breath in children at the emergency department: Variability in management in Europe
Source: PLoS One. 2021 May 5;16(5):e0251046. doi: 10.1371/journal.pone.0251046 (PMC8099081; doi:10.1371/journal.pone.0251046)
Supplement: S11 Table — (PDF) [file pone.0251046.s011.pdf]

**S11 Table. Heatmap with odds ratios of resource use, excluding patients with triage urgency “immediate”.**

**S11a. Heatmap for different ages with odds ratios of resource use, corrected for patient characteristics[#]**

| | NL tertiary | NL teaching | UK | PT | AT |
|---|---|---|---|---|---|
| Blood tests all children | 4.3* | 1.4* | + | 1.6* | 5.9* |
| < 1 year | 3.7* | 1.4** | + | 2.1* | 6.7* |
| > 1 year | 4.6* | 1.5* | + | 1.3* | 5.6* |
| X-rays all children | 4.6* | + | 2.0* | 8.5* | 3.9* |
| < 1 year | 10.7* | + | 5.2* | 18.4* | 11.8* |
| > 1 year | 3.8* | + | 1.7* | 7.1* | 3.1* |
| Inhalation medication all children | 1.1** | 1.6* | 1.8* | 2.2* | + |
| < 1 year | 1.3** | 1.9* | + | 3.1* | 1.8* |
| > 1 year | 1.4* | 1.9* | 2.6* | 2.3* | + |
| Intravenous medication all children | 1.9* | 3.9* | + | 1.0** | 1.1** |
| < 1 year | 2.5* | 4.3* | + | 1.3** | 1.9** |
| > 1 year | 1.9* | 4.3* | 1.1** | 0.9** | + |
| General admission all children | 9.3* | 7.6* | 4.0* | + | 2.2* |
| < 1 year | 6.5* | 4.6* | 1.4* | + | 1.4* |
| > 1 year | 13.0* | 11.1* | 6.9* | + | 2.9* |
| ICU admission all children | 145.6* | 2.2** | 4.2** | 16.5* | + |
| < 1 year | 215.9** | + | 1.4** | 43.2** | 3.3** |
| > 1 year | 114.8* | 2.6** | 4.8** | 7.4** | + |

[#]Associations are determined by multivariable logistic regression models. Model adjusted for sex, age, season, triage urgency, fever, tachycardia, tachypnoea, low oxygen saturation and increased work of breathing.

[+] reference. * P-value <0.01. ** not significant

NL teaching = Maasstad Hospital, Rotterdam, the Netherlands; NL tertiary = Erasmus MC, Rotterdam, the Netherlands; UK = St Mary’s Hospital, London, United Kingdom; PT = Hospital Fernando da Fonseca, Lisbon, Portugal; AT = General Hospital, Vienna, Austria.

**S11b. Heatmap for patients with different severity with odds ratios of resource use, corrected for patient characteristics#**

| | NL tertiary | NL teaching | UK | PT | AT |
|---|---|---|---|---|---|
| Blood tests all children | 4.3* | 1.4* | + | 1.6* | 5.9* |
| severe | 4.0* | 1.2** | + | 1.4* | 6.3* |
| non-severe | 14.3* | 4.6* | + | 4.3* | 11.7* |
| X-rays all children | 4.6* | + | 2.0* | 8.5* | 3.9* |
| severe | 4.2* | + | 1.9* | 7.5* | 4.1* |
| non-severe | 9.1* | + | 2.3** | 21* | 7.3* |
| Inhalation medication all children | 1.1** | 1.6* | 1.8* | 2.2* | + |
| severe | 1.2** | 1.7* | 2.1* | 2.2* | + |
| non-severe | + | 2.9* | 2.0* | 4.6* | 1.8* |
| Intravenous medication all children | 1.9* | 3.9* | + | 1.0** | 1.1** |
| severe | 2.6* | 4.0* | 1.4* | + | 2.4* |
| non-severe | 9.9* | 65.8* | + | 15.1* | 4.3** |
| General admission all children | 9.3* | 7.6* | 4.0* | + | 2.2* |
| severe | 10.3* | 6.9* | 4.1* | + | 2.4* |
| non-severe | 10.7* | 12.6* | 1.9** | + | 1.6** |
| ICU admission all children | 145.6* | 2.2** | 4.2** | 16.5* | + |
| severe | 97.5* | 1.8** | 3.2** | 11.6* | + |
| non-severe | n.a. | n.a. | n.a. | n.a. | n.a. |

#Associations are determined by multivariable logistic regression models. Model adjusted for sex, age, season, triage urgency, fever, tachycardia, tachypnoea, low oxygen saturation and increased work of breathing.

+ reference. * P-value <0.01. ** not significant

NL teaching = Maasstad Hospital, Rotterdam, the Netherlands; NL tertiary = Erasmus MC, Rotterdam, the Netherlands; UK = St Mary's Hospital, London, United Kingdom; PT = Hospital Fernando da Fonseca, Lisbon, Portugal; AT = General Hospital, Vienna, Austria.
