# Supplementary material for: Shortness of breath in children at the emergency department: Variability in management in Europe
Source: PLoS One. 2021 May 5;16(5):e0251046. doi: 10.1371/journal.pone.0251046 (PMC8099081; doi:10.1371/journal.pone.0251046)
Supplement: S12 Table — (PDF) [file pone.0251046.s012.pdf]

S12 Table. Heatmap with odds ratios of resource use, including correction for referral.

S12a. Heatmap for different ages with odds ratios of resource use, corrected for patient characteristics#

	NL tertiary	NL teaching	UK	PT
Blood tests all children	3.1*	1.0**	+	1.8*
< 1 year	2.8*	1.0**	+	2.2*
> 1 year	3.3*	1.0**	+	1.5*
X-rays all children	4.8*	+	2.6*	10.9*
< 1 year	10.4*	+	6.7*	24.2*
> 1 year	4.0*	+	2.1*	9.1*
Inhalation medication all children	+	1.3*	1.4*	1.7*
< 1 year	1.3**	1.9*	+	2.7*
> 1 year	+	1.3*	1.7*	1.5*
Intravenous medication all children	1.8*	4.1*	+	1.5*
< 1 year	2.2*	3.6*	+	1.5*
> 1 year	1.5*	4.3*	+	1.4*
General admission all children	6.7*	6.0*	3.6*	+
< 1 year	4.4*	3.5*	1.2**	+
> 1 year	8.7*	8.9*	6.1*	+
ICU admission all children	28.8*	+	1.8**	9.7*
< 1 year	47.9*	+	1.5**	43.0*
> 1 year	23.5*	+	1.6**	3.4*

#Associations are determined by multivariable logistic regression models. Model adjusted for sex, age, referral, season, triage urgency, fever, tachycardia, tachypnoea, low oxygen saturation and increased work of breathing.
+reference. * P-value <0.01. ** not significant
NL teaching = Maasstad Hospital, Rotterdam, the Netherlands; NL tertiary = Erasmus MC, Rotterdam, the Netherlands; UK = St Mary's Hospital, London, United Kingdom; PT = Hospital Fernando da Fonseca, Lisbon, Portugal.

S12b. Heatmap for patients with different severity with odds ratios of resource use, corrected for patient characteristics#

	NL tertiary	NL teaching	UK	PT
Blood tests all children	3.1*	1.0**	+	1.8*
severe	3.2*	+	1.2**	1.9*
non-severe	9.5*	3.5*	+	4.9*
X-rays all children	4.8*	+	2.6*	10.9*
severe	4.4*	+	2.5*	9.7*
non-severe	8.0*	+	2.9**	28.3*
Inhalation medication all children	+	1.3*	1.4*	1.7*
severe	+	1.3*	1.5*	1.7*
non-severe	+	3.1*	2.0*	4*
Intravenous medication all children	1.8*	4.1*	+	1.5*
severe	1.7*	3*	+	1.2**
non-severe	6.8**	57.5*	+	15.4*
General admission all children	6.7*	6.0*	3.6*	+
severe	6.8*	5.3*	3.7*	+
non-severe	7.8*	12.0*	1.9**	+
ICU admission all children	28.8*	+	1.8**	9.7*
severe	25.5*	+	1.7**	8.5*
non-severe	n.a.	n.a.	n.a.	n.a.

[#]Associations are determined by multivariable logistic regression models. Model adjusted for sex, age, referral, season, triage urgency, fever, tachycardia, tachypnoea, low oxygen saturation and increased work of breathing.
[+]reference. * P-value <0.01. ** not significant
NL teaching = Maasstad Hospital, Rotterdam, the Netherlands; NL tertiary = Erasmus MC, Rotterdam, the Netherlands; UK = St Mary's Hospital, London, United Kingdom; PT = Hospital Fernando da Fonseca, Lisbon, Portugal.
