# Supplementary material for: Shortness of breath in children at the emergency department: Variability in management in Europe
Source: PLoS One. 2021 May 5;16(5):e0251046. doi: 10.1371/journal.pone.0251046 (PMC8099081; doi:10.1371/journal.pone.0251046)
Supplement: Attachment — Submitted filename: Response to Reviewers.docx [file pone.0251046.s013.docx]

Dear Ms. Morrow, dear reviewers

Thank you for your time, effort and expertise in reviewing our manuscript and taking it under consideration for publication.

We have adapted the manuscript according to the comments and we think that the manuscript improved after these important comments.

Please find our response below in blue.

**Van:** <[em.pone.0.6e5d4a.295f74bb@editorialmanager.com](mailto:em.pone.0.6e5d4a.295f74bb@editorialmanager.com)> namens PLOS ONE <[em@editorialmanager.com](mailto:em@editorialmanager.com)>
**Beantwoorden - Aan:** PLOS ONE <[plosone@plos.org](mailto:plosone@plos.org)>
**Datum:** vrijdag 2 oktober 2020 om 10:52 AM
**Aan:** Dorine_emc <[d.borensztajn@erasmusmc.nl](mailto:d.borensztajn@erasmusmc.nl)>
**Onderwerp:** PLOS ONE Decision: Revision required [PONE-D-20-19274] - [EMID:e9b8124a2751f626]

PONE-D-20-19274
Shortness of breath in children at the emergency department: variability in management in Europe.
PLOS ONE

Dear Dr. Borensztajn,

Thank you for submitting your manuscript to PLOS ONE. After careful consideration, we feel that it has merit but does not fully meet PLOS ONE’s publication criteria as it currently stands. Therefore, we invite you to submit a revised version of the manuscript that addresses the points raised during the review process.


Please submit your revised manuscript by Nov 16 2020 11:59PM. If you will need more time than this to complete your revisions, please reply to this message or contact the journal office at [plosone@plos.org](mailto:plosone@plos.org). When you're ready to submit your revision, log on to <https://www.editorialmanager.com/pone/> and select the 'Submissions Needing Revision' folder to locate your manuscript file.

Please include the following items when submitting your revised manuscript:

- A rebuttal letter that responds to each point raised by the academic editor and reviewer(s). You should upload this letter as a separate file labeled 'Response to Reviewers'.
- A marked-up copy of your manuscript that highlights changes made to the original version. You should upload this as a separate file labeled 'Revised Manuscript with Track Changes'.
- An unmarked version of your revised paper without tracked changes. You should upload this as a separate file labeled 'Manuscript'.

If you would like to make changes to your financial disclosure, please include your updated statement in your cover letter. Guidelines for resubmitting your figure files are available below the reviewer comments at the end of this letter.

If applicable, we recommend that you deposit your laboratory protocols in protocols.io to enhance the reproducibility of your results. Protocols.io assigns your protocol its own identifier (DOI) so that it can be cited independently in the future. For instructions see: [http://journals.plos.org/plosone/s/submission-guidelines#loc-laboratory-protocols](https://eur01.safelinks.protection.outlook.com/?url=http%3A%2F%2Fjournals.plos.org%2Fplosone%2Fs%2Fsubmission-guidelines%23loc-laboratory-protocols&data=02%7C01%7Cd.borensztajn%40erasmusmc.nl%7Ca85b17fa71f34f42c51608d869c43d5a%7C526638ba6af34b0fa532a1a511f4ac80%7C0%7C0%7C637375638843837126&sdata=GokUFzr8u%2FL1Xm6iy2IUNbsQmQKt9uGsxU7JjTzNf3s%3D&reserved=0)

We look forward to receiving your revised manuscript.

Kind regards,

Brenda M. Morrow, PhD
Academic Editor
PLOS ONE

Journal Requirements:

When submitting your revision, we need you to address these additional requirements.

1. Please ensure that your manuscript meets PLOS ONE's style requirements, including those for file naming. The PLOS ONE style templates can be found at

<https://journals.plos.org/plosone/s/file?id=wjVg/PLOSOne_formatting_sample_main_body.pdf> and

<https://journals.plos.org/plosone/s/file?id=ba62/PLOSOne_formatting_sample_title_authors_affiliations.pdf>

Thank you for pointing this out and apologize for this.
We have amended our document to meet PLOS ONE’s style requirements.

2. Thank you for including your ethics statement:

"The study was approved by the institutional review boards of all participating institutions and the need for informed consent was waived.".

i) Please amend your current ethics statement to include the full name of the ethics committee/institutional review board(s) that approved your specific study.

We have amended the ethics statement and have included the full names of the ethics committees.

*“The study was approved by the medical ethical committees of the participating institutions: Medical Ethics Committee Erasmus MC (MEC-2013-567), Board of Directors Maasstad Ziekenhuis (L2013-103), Imperial College London Joint Research Compliance Office (14/WA/1051), Comissão de Ética para a Saúde do Hospital Prof. Dr. Fernando Fonseca EPE (Estudo Clínico TrIAGE – Parecer Favorável), Ethik Kommission Medizinische der Medizinischen Unversität Wien (EK Nr: 1405/2014). “*

ii) Once you have amended this/these statement(s) in the Methods section of the manuscript, please add the same text to the “Ethics Statement” field of the submission form (via “Edit Submission”).

We have added the same text to the submission form.

For additional information about PLOS ONE ethical requirements for human subjects research, please refer to <http://journals.plos.org/plosone/s/submission-guidelines#loc-human-subjects-research>.

3. We note that you have stated that you will provide repository information for your data at acceptance. Should your manuscript be accepted for publication, we will hold it until you provide the relevant accession numbers or DOIs necessary to access your data. If you wish to make changes to your Data Availability statement, please describe these changes in your cover letter and we will update your Data Availability statement to reflect the information you provide.

We will provide repository information upon acceptance of our manuscript, our institution supports the safe reposition of data and, a DOI will be provided.

4.  We note that you have included the phrase “data not shown” in your manuscript. Unfortunately, this does not meet our data sharing requirements. PLOS does not permit references to inaccessible data. We require that authors provide all relevant data within the paper, Supporting Information files, or in an acceptable, public repository. Please add a citation to support this phrase or upload the data that corresponds with these findings to a stable repository (such as Figshare or Dryad) and provide and URLs, DOIs, or accession numbers that may be used to access these data. Or, if the data are not a core part of the research being presented in your study, we ask that you remove the phrase that refers to these data.

Thank you for pointing this out. We have removed the phrase “data not shown” and have added the relevant data as appendix tables: S6 table “Differences in resource use in children with and without comorbidity“ and S7 table “Heatmap with odds ratios of resource use, excluding patients with comorbidity” for the analysis involving comorbidity and S10 table “Heatmap with odds ratios of resource use, including correction for referral” for the analysis involving referral.

5. Please include captions for your Supporting Information files at the end of your manuscript, and update any in-text citations to match accordingly. Please see our Supporting Information guidelines for more information: [http://journals.plos.org/plosone/s/supporting-information](about:blank).

We have added captions for the supporting files to the manuscript.

[Note: HTML markup is below. Please do not edit.]

Reviewers' comments:

Reviewer's Responses to Questions

**Comments to the Author**

1. Is the manuscript technically sound, and do the data support the conclusions?

The manuscript must describe a technically sound piece of scientific research with data that supports the conclusions. Experiments must have been conducted rigorously, with appropriate controls, replication, and sample sizes. The conclusions must be drawn appropriately based on the data presented.

Reviewer #1: Partly

Reviewer #2: Partly

2. Has the statistical analysis been performed appropriately and rigorously?

Reviewer #1: I Don't Know

Reviewer #2: I Don't Know

3. Have the authors made all data underlying the findings in their manuscript fully available?

The [PLOS Data policy](https://eur01.safelinks.protection.outlook.com/?url=http%3A%2F%2Fwww.plosone.org%2Fstatic%2Fpolicies.action%23sharing&data=02%7C01%7Cd.borensztajn%40erasmusmc.nl%7Ca85b17fa71f34f42c51608d869c43d5a%7C526638ba6af34b0fa532a1a511f4ac80%7C0%7C0%7C637375638843847120&sdata=%2FWSf6dxPVcChdpHJmZdJW40r%2BOXheqcmr%2BgG1LuVbgg%3D&reserved=0) requires authors to make all data underlying the findings described in their manuscript fully available without restriction, with rare exception (please refer to the Data Availability Statement in the manuscript PDF file). The data should be provided as part of the manuscript or its supporting information, or deposited to a public repository. For example, in addition to summary statistics, the data points behind means, medians and variance measures should be available. If there are restrictions on publicly sharing data—e.g. participant privacy or use of data from a third party—those must be specified.

Reviewer #1: Yes

Reviewer #2: Yes

4. Is the manuscript presented in an intelligible fashion and written in standard English?

PLOS ONE does not copyedit accepted manuscripts, so the language in submitted articles must be clear, correct, and unambiguous. Any typographical or grammatical errors should be corrected at revision, so please note any specific errors here.

Reviewer #1: Yes

Reviewer #2: Yes

5. Review Comments to the Author

Please use the space provided to explain your answers to the questions above. You may also include additional comments for the author, including concerns about dual publication, research ethics, or publication ethics. (Please upload your review as an attachment if it exceeds 20,000 characters)

Reviewer #1: This article examines the variation in management of children presenting to the emergency departments across multiple institutions. This variation could represent opportunities for standardization of management to improve patient care.

Major:
-Imputation used for missing values. RR range of completion reported 15-93% which concerns me for this to be used as one of the main variables used for an illness severity marker.

Thank you for highlighting this important issue. We agree that number range of missing variable per hospital is large, but the median of available vital signs for the total population showed adequate numbers: heart rate 93.7% (range between hospitals 91.0%-95.8%), respiratory rate 70.0% (range 15.2%-93.0%), temperature 94.9% (range 84.0%-99.7%) and oxygen saturation 97.0% (range 91.6%-99.8%).

We agree that especially respiratory rate has missing data in our study. However, this reflects routine care in many EDs and is one of the downsides of working with routine data. For example, the multicenter study of van de Maat et al. showed that complete vital sign measurement occurred in only half of pediatric ED visits and respiratory rate was missing most frequently. However, the numbers in our study, including those of other variables, are large enough to perform multiple imputation of the missing values.
When it comes to handling missing data such as vital signs in research, multiple imputation is considered to be superior to other methods and this statement is supported by several simulation studies, including simulation studies showing unbiased results even with large proportions of missing data (up to 90%), provided sufficient auxiliary information was available.
We have addressed this issue in the limitations section.
Van de Maat, 2020, PMID 32036433; Donders 2006, PMID 16980149; Hu 2017, PMID 28323112, Madley-Dowd, 2019, PMID 30878639.

*“In our study, there was a number of missing vital signs, especially respiratory rate. However, this reflects actual routine care at many EDs [49] and is one of the limitations of using routine data. For example, the multicenter study of van de Maat et al. showed that complete vital sign measurement (temperature, heart rate, respiratory rate and capillary refill) occurred in only half of pediatric ED visits and respiratory rate was the vital sign missing most frequently. [49] In our study, the median number of vital sign measurement and other clinical variables were large enough* to perform multiple imputation of the missing values. *When using routine data for research purposes, multiple imputation is considered a useful strategy for handling missing data and superior to other methods; this statement is supported by several simulation studies,* including simulation studies showing unbiased results even with large proportions of missing data, provided sufficient auxiliary information was available. *[16-18,50-51]”*

-Discussion organization can be improved, current form seems to be more of a list of findings, would be helpful to add how this compares to other studies and applying the findings to clinical practice. I would focus more on why variation matters (cost, outcomes, etc) and that guidelines have been shown to improve this.

Thank you for this valuable comment. We have expanded the discussion session and included a more elaborate discussion of the clinical implications of our study.

*“Variability in management reflects high and potentially avoidable resource use, which increases health care costs, ED crowding, adverse events and reduces quality of care. [3,33]
Reduction of this variability is a good starting point to decrease healthcare costs and improve quality of care. [33]
The use of clinical practice guidelines can potentially reduce variability and improve quality of care, [3] however, the mere availability of guidelines does not automatically entail guideline adherence. [1, 34-36] Several studies have shown that interventions to improve guideline adherence reduce resource use without negatively affecting other outcomes such as hospital admission rates. [23,37-40] Furthermore, other interventions, such as bedside viral tests or a bedside CRP test, can positively impact resources use, such, x-rays, antibiotic use and hospital admission. [22]
Our data identifies a large group of children that can benefit from adherence to guidelines and bedside tests.”*

Minor
-How were comorbidities obtained? Problem list versus billing data?
Comorbidity was documented in free text fields and coded based on the International Classification of Diseases, Ninth Revision (ICD-9 codes).

*“Comorbidity was documented in free text fields and was classified into complex comorbidity, noncomplex comorbidity, and no comorbidity based on the Paediatric Medical Complexity Algorithm.”*

- Knowing the specific respiratory past medical history would be helpful in interpreting data, especially the use of inhalation medications (i.e. asthma, BPD)
Thank you for pointing out this important point.
In two settings, no relevant numbers of patients with comorbidity were found. In the sub-analysis of children without comorbidity, the overall variability in resource use and hospitalization remained similar in the 5 EDs (S7 table).
Furthermore, we performed a sub-analysis for children with a past medical history of asthma (N = 529) in the three settings where information on comorbidity was available and have added this as analysis as an appendix (S8 table “Heatmap with odds ratios of resource use for children with a past medical history of asthma”). This analysis still shows considerable variation in resource use such as inhalation medication or lab tests. Hospital admission was not significantly different between the different emergency departments.

*“Comorbidity could explain some but not all of the variance found between hospitals. Sub-analysis of the settings with > 10% comorbidity (Vienna, London and the tertiary hospital in Rotterdam), showed higher resource use in children with comorbidity, especially in children with complex comorbidity (S6 table). In the sub-analysis of children without comorbidity, the overall variability in resource use and hospitalization remained similar in the 5 EDs (S7 table).
Furthermore, a sub-analysis of children with a past medical history of asthma, (three settings, N = 529) still shows considerable variation in resource use, including the use of inhalation medication or and lab tests (S8 table).”*

-How was increased work of breathing measured? Subjective by provider versus scoring system, if the latter is it a validated tool?
Increased work of breathing was measured as a discriminator of the flowchart “shortness of breath” of the Manchester Triage System (MTS) defined as “increased respiratory rate, use of accessory muscles and grunting”, in which all nurses received formal standardized training according to the MTS guideline.
Emergency Triage: Manchester Triage Group, 2nd Edition. Kevin Mackway-Jones (Editor), Janet Marsden (Editor), Jill Windle (Editor), ISBN: 9780727915429, 2006.

-Were vital signs recorded just initial or ongoing?
For vital signs, the first measurement at triage was used for analysis.

-What is electrical therapy? (line 181)
electrical therapy is defined as defibrillation, emergent cardioversion, or external pacing (S2 table “Immediate life-saving interventions”).

-Results: Good sample size overall, but almost half of encounters were from same hospital
We agree that as the Portuguese hospital has a larger number of annual visits, a relatively large part of all encounters were from this hospital, however, all hospitals provided over 1000 inclusions. We performed multivariable logistic regression analysis in which we used hospital setting as a factor.

-Statement majority had abnormal respiratory rate, but <50% at each site reported to be tachypnic in table 1

Thank you for noticing this mistake, we have corrected the rates of tachypnea in the different hospitals.

*“The rate of tachypnoea varied between 3.9% in Austria and 49.2% in the UK.”*

-abstract “categorized as very urgent ranging from 13-80%" (but in results you give 12.1 and 77% as the range)
the very urgent referred to in the abstract is the combined MTS category emergent/very urgent, and have clarified this in the abstract and have combined these categories in table 1 as well for clarification.

*“Of those, 7,379 were categorized as MTS categories immediate/very urgent, ranging from 13-80% in the participating hospitals.”*

- Alternate between commas and periods used as decimal points in text and tables
We have corrected this issue.

- Line 264-265 add p-value for sub-analysis
We have added this data (S6 table “Differences in resource use in children with and without comorbidity” and S7 table “Heatmap with odds ratios of resource use, excluding patients with comorbidity”).

-Limitations that should be highlighted: missing factors noted to impact resource use in other studies not included in this study, unable to account for relevant pmh specific to management approach, unknown what testing/management prior to arrival for referred patients
Thank you for addressing this issue. We have added these factors to the limitation section

*“Although we adjusted for patient characteristics and markers of disease severity, we cannot exclude that unmeasured aspects of patient or setting characteristics, such as past medical history, tests or interventions performed prior to arrival at the ED, immunization status or physician experience and background, availability of primary care could still be of influence.”*

-appendix 1 include p-values comparing sites.
Thank you for this suggestion. We thought it would be more useful to add p-values to table 1 than to appendix 1, as both compare sites, but table 1 compares sites on a patient level and thus contains more measurements than appendix 1, which compares sites on a setting level and therefore only contains 5 measurements (5 settings). Due to these small numbers, we did not perform statistical analysis on this level, however, we have added the information on comorbidity to table 1 to be able to compare settings on this variable as well.

What does the estimation annotation mean in S1 Table “Description of the different study sites”?
In one or two settings, data on self-referral (1 setting) and comorbidity (2 settings) was available on a setting level but unfortunately not an individual patient level. We have clarified this in S1 Table “Description of the different study sites”.

*“* overall data for this setting, not available for each individual patient”*

Reviewer #2: This is an interesting study focused on a different way of approaching the evaluation of variability in healthcare settings. This focuses on the chief complaint of shortness of breath as opposed to diagnosis-based assessment of variability. However, they did perform statistical analysis to try to adjust for the diversity of patient population both in acuity and other possible confounding factors. The triage process was standardized throughout hospitals.

Consider removing Triage level 1 acuity (highest acuity) as this population likely should not be a population that decreases variation, in fact most patients in this category likely get a large amount of resource utilization which is likely appropriate.

Thank you for your suggestion. We have performed a sub-analysis excluding children with level 1 acuity of the MTS and added this as an appendix. (S9 table “Heatmap with odds ratios of resource use, excluding patients with triage urgency “immediate”). The results were similar before and after excluding the group of children with highest acuity, showing large variability in all types of resource use.

*“When performing a separate analysis after removing patients with triage urgency immediate (the highest acuity group), similar trends with high variability in all types of resource use were seen (S9 table).”*

With the analysis did you cluster by hospital center?

We did not perform multilevel analysis and as such did not cluster by hospital center, as several authors do not recommend multilevel analysis with a group size of only 5 settings.
However, we did perform multivariable logistic regression analysis in which we used hospital setting as a factor.
Moineddin 2007, PMID 17634107; Ali, 2019, PMID 31756205.

I have some concerns that the respiratory rate was missing most frequently (70% of the time, but down to 15% at its worst) in a manuscript that is addressing variability in patient presenting with shortness of breath. Can you explain this?

Thank you for highlighting this important issue. We agree that number range of missing variable per hospital is large, but the median of available vital signs for the total population showed adequate numbers: heart rate 93.7% (range between hospitals 91.0%-95.8%), respiratory rate 70.0% (range 15.2%-93.0%), temperature 94.9% (range 84.0%-99.7%) and oxygen saturation 97.0% (range 91.6%-99.8%).

We agree that especially respiratory rate has missing data in our study. However, this reflects routine care in many EDs and is one of the downsides of working with routine data. For example, the multicenter study of van de Maat et al. showed that complete vital sign measurement occurred in only half of pediatric ED visits and respiratory rate was missing most frequently. However, the numbers in our study are large enough to perform multiple imputation of the missing values.
When it comes to handling missing data such as vital signs in research, multiple imputation is considered to be superior to other methods and this statement is supported by several simulation studies, including simulation studies showing unbiased results even with large proportions of missing data (up to 90%).
We have addressed this issue in the limitations section.
Van de Maat, 2020, PMID 32036433; Donders 2006, PMID 16980149; Hu 2017, PMID 28323112, Madley-Dowd, 2019, PMID 30878639.

*“In our study, there was a number of missing vital signs, especially respiratory rate. However, this reflects actual routine care at many EDs [49] and is one of the limitations of using routine data. For example, the multicenter study of van de Maat et al. showed that complete vital sign measurement (temperature, heart rate, respiratory rate and capillary refill) occurred in only half of pediatric ED visits and respiratory rate was the vital sign missing most frequently. [49] In our study, the median number of vital sign measurement and other clinical variables were large enough* to perform multiple imputation of the missing values. *When using routine data for research purposes, multiple imputation is considered a useful strategy for handling missing data and superior to other methods; this statement is supported by several simulation studies,* including simulation studies showing unbiased results even with large proportions of missing data*,* provided sufficient auxiliary information was available. *[16-18,50-51]”*

I think my largest concern is related to whether or not your dataset has data related to patient diagnosis to be able to adjust for differences related to a patient resource utilizations with pneumonia versus asthma. As those two presentations although may have the same chief complaint, they likely require two different workups and are based on an provider assessment.

We thank the reviewer for raising this important point, but unfortunately, we don’t have this information available for analysis. It is of course true that pneumonia should be managed differently than asthma (e.g. inhalation medication is appropriate in asthma but not in pneumonia). At the ED final diagnoses are missing in many cases and diagnostics and interventions are based on a working diagnosis. Discrimination between different cause of respiratory illness can be difficult. Despite this lack of diagnosis and differences in management per diagnosis, there is also considerable overlap in the management of children with respiratory complaints regardless the final diagnosis. Except for the Portuguese setting, which recommends lab tests and x-rays in children with suspected bacterial infection, the national guidelines of all other participating settings state that resource use such as imaging or lab tests are not recommended except in severe cases of asthma, bronchiolitis or pneumonia. Despite this advice, we still found high resource use and high variation in the severe as well as the non-severe group in our study.
Furthermore, inhalation medication is not recommended in children with respiratory complaints and wheezing below the age of 1 year, as these complaints are usually caused by bronchiolitis, and there is no evidence of a positive effect of inhalation medication in this age group. However, we still found high use and high variation of inhalation medication in this specific age group between hospitals (Table 2 “Heatmap for different ages with odds ratios of resource use, corrected for patient characteristics”).
Yusuf, 2019, PMID 31803260.

We have highlighted this issue in the discussion as follows:

*“We used presenting symptom as the main inclusion criterion and not final diagnosis, which is usually not available in the early phase of the diagnostic process. This resulted in a heterogeneous study population of children with different final diagnoses, such as upper respiratory tract infections, viral and bacterial lower respiratory tract infections, viral induced wheezing and asthma. Although our study included children with these different final diagnoses, and in part management depends on the final diagnosis, there is also considerable overlap in the management of these diagnoses.*

*At the ED final diagnoses are missing in many cases and diagnostics and interventions are based on a working diagnosis. Discrimination between different cause of respiratory illness can be difficult. Participating countries’ national guidelines regarding the main final diagnoses of children visiting the ED with shortness of breath, such as asthma, bronchiolitis and pneumonia, clearly state that diagnostic tests such as blood tests or x-rays have no place in the assessment of children with any of these diagnoses, except in atypical or severe cases (e.g. children that require PICU admission), [43-46] except the National Portuguese guidelines [47] which advice to perform lab tests in all children with suspected bacterial disease and X-rays in children with bronchiolitis with suspected bacterial infection. Although this might explain the high rate of X-rays that were seen in the Portuguese setting, our data still showed large variation in resource use and hospitalization in the severe as well as the non-severe group of children with respiratory symptoms that cannot be explained by guideline content. Furthermore, inhalation medication is not recommended in children with respiratory complaints and wheezing below the age of 1 year, as these complaints are usually caused by bronchiolitis, and there is no evidence of a positive effect of inhalation medication in this age group. However, we still found high use and high variation of inhalation medication in this specific age group. [48] Lastly, hospital admission in general is guided by disease severity and not by final diagnosis. [43-47] “*

You should consider that the tertiary center may have increased use of the ICU due to transfers from outside hospitals and have higher acuity patients. Did you account for interhospital transfers?

The ICU admission rate does not include interhospital transfers, as these children are admitted to the ICU directly and bypass the ED.

6. PLOS authors have the option to publish the peer review history of their article ([what does this mean?](https://eur01.safelinks.protection.outlook.com/?url=https%3A%2F%2Fjournals.plos.org%2Fplosone%2Fs%2Feditorial-and-peer-review-process%23loc-peer-review-history&data=02%7C01%7Cd.borensztajn%40erasmusmc.nl%7Ca85b17fa71f34f42c51608d869c43d5a%7C526638ba6af34b0fa532a1a511f4ac80%7C0%7C0%7C637375638843847120&sdata=XtliwIp3sEeNTUtJMRa%2BNktFRTeggW1FUmrlZKFaomY%3D&reserved=0)). If published, this will include your full peer review and any attached files.


If you choose “no”, your identity will remain anonymous but your review may still be made public.


**Do you want your identity to be public for this peer review?** For information about this choice, including consent withdrawal, please see our [Privacy Policy](https://eur01.safelinks.protection.outlook.com/?url=https%3A%2F%2Fwww.plos.org%2Fprivacy-policy&data=02%7C01%7Cd.borensztajn%40erasmusmc.nl%7Ca85b17fa71f34f42c51608d869c43d5a%7C526638ba6af34b0fa532a1a511f4ac80%7C0%7C0%7C637375638843857114&sdata=9EGRUk1fSfhwgq4mFr0gWKYATAFmtco2UHd8HPsOjVE%3D&reserved=0).

Reviewer #1: **Yes:** Aleisha M Nabower

Reviewer #2: No

[NOTE: If reviewer comments were submitted as an attachment file, they will be attached to this email and accessible via the submission site. Please log into your account, locate the manuscript record, and check for the action link "View Attachments". If this link does not appear, there are no attachment files.]

While revising your submission, please upload your figure files to the Preflight Analysis and Conversion Engine (PACE) digital diagnostic tool, [https://pacev2.apexcovantage.com/](https://eur01.safelinks.protection.outlook.com/?url=https%3A%2F%2Fpacev2.apexcovantage.com%2F&data=02%7C01%7Cd.borensztajn%40erasmusmc.nl%7Ca85b17fa71f34f42c51608d869c43d5a%7C526638ba6af34b0fa532a1a511f4ac80%7C0%7C0%7C637375638843857114&sdata=jQv%2BEoWyi6di7Let%2BdhTQBqYCw4pYAo5jUGi02%2BZvzo%3D&reserved=0). PACE helps ensure that figures meet PLOS requirements. To use PACE, you must first register as a user. Registration is free. Then, login and navigate to the UPLOAD tab, where you will find detailed instructions on how to use the tool. If you encounter any issues or have any questions when using PACE, please email PLOS at [figures@plos.org](mailto:figures@plos.org). Please note that Supporting Information files do not need this step.

*In compliance with data protection regulations, you may request that we remove your personal registration details at any time.* [*(Remove my information/details)*](https://eur01.safelinks.protection.outlook.com/?url=https%3A%2F%2Fwww.editorialmanager.com%2Fpone%2Flogin.asp%3Fa%3Dr&data=02%7C01%7Cd.borensztajn%40erasmusmc.nl%7Ca85b17fa71f34f42c51608d869c43d5a%7C526638ba6af34b0fa532a1a511f4ac80%7C0%7C0%7C637375638843867112&sdata=%2Fv9p37kL%2B9RsbJ7%2F7RT8GCLgHM9UsqJ85lGH05c8%2Btg%3D&reserved=0)*. Please contact the publication office if you have any questions.*
