# Supplementary material for: Shortness of breath in children at the emergency department: Variability in management in Europe
Source: PLoS One. 2021 May 5;16(5):e0251046. doi: 10.1371/journal.pone.0251046 (PMC8099081; doi:10.1371/journal.pone.0251046)
Supplement: Attachment — Submitted filename: rebuttal.plosone.docx [file pone.0251046.s014.docx]

Reviewer #2: I appreciate the authors addressing many of the questions that I raised. My one major concern still surrounds the following:
1) Use of the multiple imputation methodology in a manuscript describing variability in approach to shortness of breath with the vital sign that is missing most being the respiratory rate. Multiple imputation seems to be a helpful tool but I worry when it is replacing missing data within a primary variable (respiratory rate) related to the manuscripts main outcome. While I recognize that this data may not exist, I worry about making conclusions based on this methodology. You could consider removing the hospital that has only 15% of the respiratory rate vital sign present with the hopes that you increase the median enough to make this more valid and hopefully supporting an accurate conclusion.

We thank the reviewer for highlighting this issue and agree that including data from a setting with a large number of missing data of a primary variable, needs further explanation and might have biased the results.

First, vital signs were imputed based on the total visits of all ED’s; in this total population respiratory rate was available in 70% of the cases. Furthermore, work of breathing, which is an important marker for respiratory rate, was available for all patients. We realize that we did not mention in the methods section that work of breathing was one of the variables that was used in the imputation model and we think this supports the validity of our imputation strategy.

Second, as the reviewer suggested, we performed two additional sub-analyses.

1. Complete case analyses with complete data of respiratory rate in all settings and have found variation to be similar to the original analysis.
2. We have repeated the analysis while excluding the setting with the largest number of missing values for respiratory rate and have found variation to be similar in the remaining dataset of 4 hospitals compared to the original data set of 5 hospitals. Respiratory rate in the remaining set was available in 88% of the cases (range between settings 69-93%).

We have modified the following text to the methods section (“data analysis”):

*“The imputation model included age, sex, type of referral, MTS flowchart and urgency, time of arrival, vital sign values, work of breathing, capillary refill, level of consciousness, diagnostic tests, medication, oxygen therapy, life-saving interventions and disposition of all hospitals combined.”*

Furthermore, we have added the following text to the result section (“practice variation between hospitals”):

*“To reduce the impact of the large number of missing values for respiratory rate in one setting, two additional analyses were performed. First, a sub-analysis excluding the setting with a large number of missing values for respiratory rate was performed, which showed similar variation between the remaining four EDs compared to the original analysis (S9 table). Second, a complete case analysis for respiratory rate was performed, which showed similar variation compared to the original analysis (S10 table).”*

Lastly, we have added the following text to the discussion (“strengths and limitations”):

*“Furthermore, we performed two sub-analyses, excluding the setting with the largest number of missing values for respiratory rate, and a complete case analysis for respiratory rate and found similar variation between ED’s in comparison to the results of the original imputed dataset.”*
